# The international experience of in-situ recovery of the DCD heart: a multicentre retrospective observational study

**DOI:** 10.1016/j.eclinm.2023.101887

**Published:** 2023-03-02

**Authors:** John Louca, Marco Öchsner, Ashish Shah, Jordan Hoffman, Francisco González Vilchez, Iris Garrido, Mario Royo-Villanova, Beatriz Domínguez-Gil, Deane Smith, Leslie James, Nader Moazami, Filip Rega, Janne Brouckaert, Johan Van Cleemput, Katrien Vandendriessche, Vincent Tchana-Sato, Diawara Bandiougou, Marian Urban, Alex Manara, Marius Berman, Simon Messer, Stephen Large, WISPG, Nirav Patel, Rohan Sanghera, Constantinos Kapetanos, Antonio Rubino, Sai Bhagra, Luis-Alberto Martinez-Marin, Jordan Allen, Chindu John, Daniel Normington, Steven Tsui, Aravinda Page, Vanessa Chow, William McMaster, Alicia Pérez-Blanco, Elisabeth Torres, José Cuenca, Fernando Mosteiro, Marta Farrero, Elena Sandoval, Manuela Camino, Juan Jáurena, Fabrizio Sbraga, Eva Oliver, Antonio Quintana, Vincente Morant, Belen Estébanez, Álvaro Rocafort, Manuel Cobo, Francisco Nistal, Manuel Gómez-Bueno, Marina Pérez-Redondo, Arne Neyrinck, Diethard Monbaliu, Laurens Ceulemans

**Affiliations:** a University of Cambridge School of Clinical Medicine, Addenbrooke's Hospital, Hills Rd, Cambridge CB2 0SP, UK; b Vanderbilt Heart Transplant Unit 1215, 21st Ave, Nashville, Tennessee 37232, USA; c Spanish Registry on Heart Transplantation, Madrid, Spain; d Hospital Universitario Virgen de La Arrixaca, Ctra. Madrid-Cartagena, s/n, El Palmar, Murcia 30120, Spain; e Organización Nacional de Trasplantes, Madrid, Spain; f Department of Cardiothoracic Surgery, Langone, 1300 Franklin Avenue, Suite ML-2, Garden City, NY, USA; g Departments of Cardiac Surgery and Cardiology, The University Hospital Leuven, Leuven, Belgium; h Department of Cardiovascular Surgery, CHU Liege, Liege, Belgium; i Department of Cardiothoracic Surgery, University of Nebraska Medical Centre, 2410 Atherholt Road, Omaha, NE, USA; j The Intensive Care Unit, Southmead Hospital, North Bristol NHS Trust, Bristol BS 10 5NB, UK; k Royal Papworth Hospital Biomedical Campus, Cambridge, CB2 0AY, UK; l Golden Jubilee Hospital, Agamermnon Street, Glasgow G81 4DY, UK; m Worldwide In-Situ Perfusion Group

**Keywords:** In-situ perfusion, Thoraco-abdominal nromothermic regional perfusion, taNRP, DCD, Non heart beating donation, Donation after circulatory determination of death, DBD, Donation after brain death, Donation after neurological death, Heart beating donation, ESMP, Ex-situ machine perfusion, CS, Cold storage

## Abstract

**Background:**

Heart transplantation is an effective treatment offering the best recovery in both quality and quantity of life in those affected by refractory, severe heart failure. However, transplantation is limited by donor organ availability. The reintroduction of heart donation after the circulatory determination of death (*DCD*) in 2014 offered an uplift in transplant activity by 30%. Thoraco-abdominal normothermic regional perfusion (taNRP) enables in-situ reperfusion of the DCD heart. The objective of this paper is to assess the clinical outcomes of DCD donor hearts recovered and transplanted from donors undergoing taNRP.

**Method:**

This was a multicentre retrospective observational study. Outcomes included functional warm ischaemic time, use of mechanical support immediately following transplantation, perioperative and long-term actuarial survival and incidence of acute rejection requiring treatment. 157 *taNRP* DCD heart transplants, performed between February 2, 2015, and July 29, 2022, have been included from 15 major transplant centres worldwide including the UK, Spain, the USA and Belgium. 673 donations after the neurological determination of death (DBD) heart transplantations from the same centres were used as a comparison group for survival.

**Findings:**

taNRP resulted in a 23% increase in heart transplantation activity. Survival was similar in the taNRP group when compared to DBD. 30-day survival was 96.8% ([92.5%–98.6%] 95% CI, n = 156), 1-year survival was 93.2% ([87.7%–96.3%] 95% CI, n = 72) and 5-year survival was 84.3% ([69.6%–92.2%] 95% CI, n = 13).

**Interpretation:**

Our study suggests that taNRP provides a significant boost to heart transplantation activity. The survival rates of *taNRP* are comparable to those obtained for DBD transplantation in this study. The similar survival may in part be related to a short warm ischaemic time or through a possible selection bias of younger donors, this being an uncontrolled observational study. Therefore, our study suggests that *taNRP* offers an effective method of organ preservation and procurement. This early success of the technique warrants further investigation and use.

**Funding:**

None of the authors have a financial relationship with a commercial entity that has an interest in the subject.

**Research in context**

**Evidence before this study**

We searched PubMed with the terms ‘taNRP’, ‘thoraco-abdominal normothermic regional perfusion’, ‘donation after circulatory death’, ‘donation after cardiac death’, ‘non heart beating donor’, ‘in-situ perfusion’ and ‘heart transplantation’. DCD transplantation with direct procurement has shown its efficacy in the last few years. Outcomes of DCD transplantation are comparable to DBD transplantation. There have been 4 clinical case reports, as well as 5 centres detailing their early experiences of taNRP. These reports have been positive, however they have not been adequately powered and have often lacked the use of a comparison group.

**Added value of this study**

This is the first, large scale, multi-centre, international case series reporting on long term (≥5 years) outcomes of taNRP. This study highlights the utility of taNRP in cardiac transplantation and the comparable outcomes to DBD. Importantly there are no significant differences in survival between ESMP and CS in our dataset, which may pave the way to a more effective, less expensive method of transplantation. However, we acknowledge that the usage of ESMP was limited compared to CS and confined to only two centres.

**Implications of all the available evidence**

The published results of taNRP are encouraging. taNRP has the potential to significantly increase the number of transplants being performed and reduce waiting list mortality. It is therefore of vital importance that we aim to further integrate it into standard clinical practice.

## Introduction

Heart transplantation (*HT*) is reserved for patients with minimal co-morbidity and end-stage heart failure (ESHF), defined as *NHYA III/IV* which is refractory to medical treatment. It is the last bastion for these patients. It offers them a greatly improved prognosis and quality of life. However, transplantation is limited by donor organ availability. This problem is related to an imbalance in demand and supply of useable donor hearts. This is despite the increasing adoption of more marginal donor organs in respect of age, cold ischaemic time, pre-donation cardiac arrest, degree of inotropic support, donor substance abuse and LV hypertrophy. More recently, additional approaches are utilising hepatitis C positive organs and donation after the circulatory determination of death (DCD).^1^^,^^2^ In addition, the use of Ex-Situ Machine Perfusion (ESMP) (*Organ Care System (OCS) developed by Transmedics*) of donor hearts has been trialled with the objective to improve donor quality and thus expand the donor pool.

In recent years the reintroduction of DCD, since it was first performed in 1967, has shown potential to increase the size of the donor organ pool. It is estimated that DCD could increase the number of heart transplantations performed by 30%.^3^ Data from 20/21 in the UK shows that DCD transplantations made up only 12% of total cardiac transplantations. However, in the Royal Papworth Hospital, DCD transplantation makes up 30% of the total number of heart transplants.^4^ Similarly, data from the US shows that in 20/21, DCD made up 5.4% of transplants,^5^ however it has been estimated that DCD could increase the donor pool by up to 30% if widely adopted.^6^ Therefore DCD transplantation and specifically taNRP have great potential in decreasing waiting list mortality.

Most hearts donated for transplantation are recovered from donors confirmed dead using neurological criteria (DBD).^3^^,^^7^ DCD donation occurs after the withdrawal of life sustaining treatment (WSLT) in a donor who does not meet the criteria for the neurological determination of death. Death is confirmed following a period of observation of complete absence of circulation and respiration, usually of 5 min duration. This time period is chosen because there is no patient that has recovered spontaneous circulation after this point, thus confirming permanent cessation of circulation.^8^

These ischaemic DCD hearts are then either reperfused outside the donor's body (*ex-situ reperfusion*) on the OCS or reperfused within the donor's body by *in-situ* reperfusion using thoraco-abdominal normo-thermic reperfusion (*taNRP*). In some cases, the heart may be preserved by both the use of *in-situ* and *ex-situ* perfusion. Direct procurement and preservation (DPP) followed by mounting of the DCD heart onto an *ex-situ* perfusion machine takes more time than restarting the circulation in situ. This further prolongs the donor heart functional total ischaemic time (FTIT). Myocardial ischaemia is the main obstacle in DCD organ transplantation. During warm ischaemia the heart is active and depletes its intracellular energy stores rapidly. The mechanisms of this ischaemic and subsequent reperfusion injury are well established.

After death is confirmed, a median sternotomy is performed and the systemic venous and arterial systems are cannulated and restoration of thoraco-abdominal flow leads to prompt termination of intra-thoracic and abdominal organ ischaemia. The aortic arch arteries are occluded before systemic perfusion occurs to prevent cerebral blood flow. After 20 min of machine perfusion, heart function is sufficiently recovered to permit weaning of *taNRP*. *Hearts are assessed by transoesophageal echocardiogram.* Moreover, this time period of 20 min is based off work done in pig hearts, which demonstrate substantial (but not total) recovery of the metabolic state of the heart after warm ischaemia within 20 min of reperfusion.^9^ Donor heart function can be assessed in this, now heart beating donor. *taNRP* significantly shortens the cardiac ischaemic time and potentially reverses the risk of permanent ischaemic damage. Results from abdominal normothermic regional perfusion (aNRP) in liver transplantation have shown significant reduction of biliary complications, graft loss and improved overall survival, when compared to the standard rapid recovery technique.^10^^,^^11^ Furthermore, results from the use of aNRP in kidney transplantation is encouraging with delayed onset of graft dysfunction and increased average utilisation of organs overall compared to rapid recovery.^12^^,^^13^ Early work on taNRP is encouraging with centres across numerous countries reporting positive outcomes.^14^, ^15^, ^16^, ^17^, ^18^, ^19^, ^20^ However, the impact of taNRP on the outcomes of DCD donor hearts has only been described in single centre experiences with small sample sizes.

Here we present the mid-long term international outcome data of 157 *taNRP* DCD heart transplants from 15 centres across the UK, Belgium, Spain and the USA. In addition, we perform a sub analysis comparing hearts that were preserved with ESMP compared to those preserved with cold storage (CS).

## Methods

### Study design

This study was a retrospective international multicentre retrospective observational study. Data was collected from 15 major transplant centres. See Supplementary Information for the full list of centres. Between February 2, 2015, and July 29, 2022 patients were enrolled and followed up. Data was collected from pre-operative and intraoperative records and collated in each centre before then being analysed.

The pre-operative parameters analysed included: donor age, donor height, donor weight, donor left ventricular ejection fraction (LVEF), recipient age and the degree of pre-operative pharmacological support. The intraoperative parameters analysed included: time from WLST (withdrawal of life supporting treatment) to reperfusion, time from WLST to the onset of FWIT (functional warm ischaemic time), FWIT to reperfusion time, recipient pulmonary vascular resistance and the duration of cold ischaemia. Post-transplantation parameters analysed included: the use of mechanical circulatory support (MCS), survival, hours spent on a ventilator, length of stay in ICU and in hospital, acute rejection warranting treatment, incidence of coronary artery vasculopathy (CAV), RV and LV function and the number of other organs transplanted. In addition to these, we compared outcomes between hearts that were preserved with ESMP compared to conventional CS.

### Patient selection

In the US, Belgium and Spain all DCD hearts were recovered using taNRP as is hospital policy in participating centres. The donors were all Maastricht category III donors. That is to say, that they were all controlled DCD donors. In the UK taNRP was only utilised in hearts donated in three centres – Addenbrookes Hospital, The Royal Papworth Hospital and Norfolk & Norwich University Hospital. Other centres in the UK were not permitted to utilise taNRP and these hearts were procured by DPP and are not explored further, as only one centre performed DPP.

We used DBD as a comparison group, because this is the gold-standard technique for treating ESHF, and it enabled us to compare the survival outcomes of taNRP. Moreover, it takes into account centre variation around survival for both techniques. DBD cases from each centre were in the time period from the first taNRP case in each centre until the censor date.

### The procedure

**Fig. 1:**
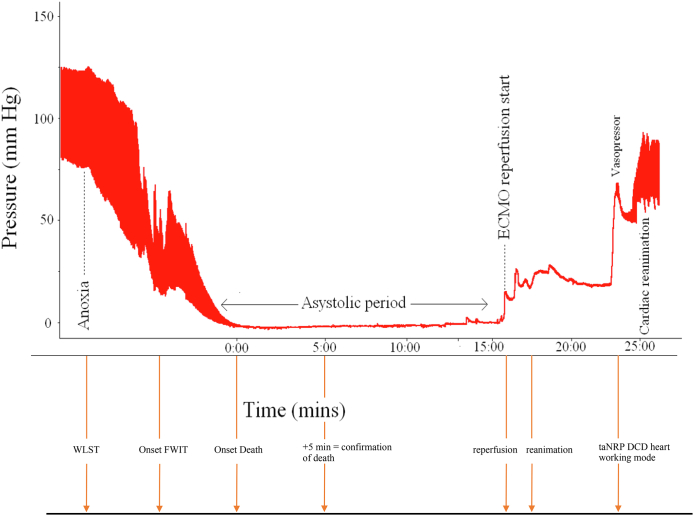
**Donor systolic blood pressure during taNRP recovery of the heart in a rat model.** The series of events is as follows. The decision is made to withdraw life sustaining treatment (WSLT). The onset of FWIT occurs when the systolic blood pressure <50 mmHg and therefore inadequate to properly perfuse the heart. The onset of death occurs when the heart first becomes asystolic. Death is confirmed after 5 min of complete absence of circulation. At this point the right atrium and the aorta are cannulated such that reperfusion can begin. The heart begins to beat shortly after.

The recovery of DCD hearts by taNRP is represented in Fig. 1.

### The use of ESMP vs. CS

The donor hearts preserved by ESMP were predominantly transplanted in the UK, with 19 of the 21 cases performed in the Royal Papworth Hospital, UK. The additional 2 hearts were from UNMC, US. In the UK, it was policy to preserve all hearts with ESMP. However, 4 hearts were preserved with CS due to the fact that the donors were in the same hospital as the recipient and therefore there was no need for the use of ESMP, due to the short ischaemic times. In UNMC the 2 hearts were preserved with ESMP because of the expected cold ischemia time greater than 4 h (distance between recovery and implant centre greater than 1000 nautical miles). All hearts from Belgium, Spain and Vanderbilt, US, NYU, US and the remaining 10 hearts from UNMC were preserved with CS. This decision was made in part for financial reasons. At NYU all donors and recipients were co-located and the heart was briefly placed in CS. It is worth noting that the taNRP protocol is the same between ESMP and CS groups. It is only after cardiac function has been restored and assessed, that the heart is then procured and subsequently either placed on ice (i.e. CS) or onto a machine to perfuse the heart (i.e., ESMP).

FTIT – Functional Total Ischaemic Time - Time of ischaemia from systolic blood pressure <50 mmHg until reperfusion.

FWIT – Functional Warm Ischaemic Time - Time of ischaemia from systolic blood pressure <50 mmHg until cold cardioplegia.

FCIT – Functional Cold Ischaemic Time - time of cold cardioplegia until reperfusion ex-situ.

Cold Ischaemic Time – Time of donor cross-clamp to reperfusion in the recipient. This only applies to hearts preserved with CS after recovery.

Therefore, in taNRP FWIT is the same as FTIT.

Additional information on the centres involved, the definition of high-volume centres and method of cannulation can be found in the supplementary information.

### Statistical analysis

Continuous data with normal distribution are expressed with means and standard deviations, whereas continuous data with a non-normal distribution are presented with medians and interquartile ranges (IQR). Analysis was performed using the pandas library v. 1.4.5 in Python 3.8.2.^21^ The association between transplantation method and survival was estimated using hazard ratios generated with a Cox Proportional-Hazards model implemented with the coxph function from the R library survival v. 3.4-0.^22^ We constructed two models, the first an unadjusted analysis without potential confounders, the second an analysis adjusted for donor and recipient ages, and cold ischaemic times, as these factors are reported to have the greatest impact on heart transplant survival. Timepoint survival probability estimates were calculated using the log (−log) transformation of the Kaplan-Meir survival curve as implemented in the lifelines library, and group-comparisons for these were performed using the log-rank test from the lifelines library in Python.^23^ The same method was used to compare CS and ESMP groups, with other group comparisons using the Wilcoxon rank sum test for continuous data and the Fisher's exact test for categorical data. Statistical significance for the primary outcome was set at a 5% level.

### Ethics approval

All centres in the study had ethical approval from their ethics departments to take part in the study. The data were anonymous and the need for informed consent was therefore waived.

### Role of the funding source

There was no funding source for this study. All authors could access the dataset and made the decision jointly to submit the paper.

## Results

This study included 157 patients who underwent taNRP transplantation and 673 patients who underwent DBD transplantation.

### Donor characteristics of taNRP & DBD groups

The median donor age was 32 years (IQR = 23–43). Of the 157 taNRP donors 26 (16.6%) were female and 131 were male (83.4%). 226 (33.6%) of donors were female in the DBD group and 447 (63.4%) were male (Table 1). This difference was significant between the two groups (p = 0.001). The mean height was 173.6 cm and mean weight was 81.6 kg in the taNRP group. The mean LV ejection fraction at the time of donation (before WSLT) was 63.3% (n = 147). 62.2% of patients were on no pre-operative pharmacological support in the form of vasoactive drugs, 22.4% were on 1 drug, 3.2% were on 2 drugs, 5.1% were on 3 drugs and 7.1% ≥4 drugs (Table 2).

**Table 1:** Comparing demographics of taNRP and DBD cases.

	taNRP	n	DBD	n	Test statistic	p-value
Donor age, years, median (IQR)	32.0 [23.0, 43.0]	157	36.0 [27.0, 46.0]	673	62,088	0.001
Donor sex, F/M, (% Female)	26/131 (16.6%)	157	226/447 (33.6%)	673	0.393	<0.001
Recipient age, years, median (IQR)	56.0 [48.0, 63.0]	157	54.0 [44.0, 62.0]	673	47,985	0.073
Recipient sex, F/M, (% Female)	34/123 (21.7%)	157	184/489 (27.3%)	673	0.735	0.159
Cold ischaemic time minutes, mean (SD)	144.2 ± 71.9	136	178.5 ± 57.8	670	55,475	0.0001
Mechanical Circulatory Support (MCS) after transplantation (total number of patients %)	20 (12.8%)	156	85 (12.7%)	670		1
IABP (intra-aortic balloon pump)	13 (8.3%)	156	15 (2.2%)	670		<0.001
ECMO (extracorporeal membrane oxygenation)	9 (5.7%)	156	36 (5.4%)	670		0.845
VAD (ventricular assisted device)	0	156	34 (5.1%)	670		<0.001
Survival at timepoints						
30 day survival	0.968	152	0.945	635	1.29	0.26
1 year survival	0.932	72	0.899	424	1.50	0.22
5 years survival	0.843	13	0.783	71	0.77	0.38

**Table 2:** Summary of taNRP donor characteristics and recipient outcomes.

Outcome	n	
Donor characteristics		
Age, years, median (IQR)	157	32.0 [23.0, 43.0]
Height (mean ± std)	157	173.6 ± 17.8
Weight (mean)	156	81.6 ± 22.1
Ejection Fraction	147	63.3 ± 6.3
Pre-operative pharmacological support in the form of vasoactive drugs	156	
No pharmacological support		97 (62.2%)
1 drug		35 (22.4%)
2 drugs		5 (3.2%)
3 drugs		8 (5.1%)
≥4 drugs		11 (7.1%)
Recipient age	157	56.0 [48.0, 63.0]
Intraoperative parameters		
Mean withdrawal to reperfusion time (std) (min)	148	26.7 ± 14.5
Mean withdrawal to FWIT (std) (min)	150	10.0 ± 12.4
Mean FWIT to reperfusion (std) (min)	148	16.7 ± 9.3
Recipient Pulmonary Vascular Resistance (mmHg/lmin-1)	144	2.6 ± 6.7
Cold Ischaemic times	136	144.2 ± 71.9
Post-transplant outcomes		
All cause mortality		
Post-transplant mortality		
30 day survival [n = at risk]	156	96.8%
1 year survival [n = at risk]	72	93.2%
5 year survival [n = at risk]	13	84.3%
Cumulative survival after taNRP heart transplantation (years)		247
Ventilation, hours, median (IQR)	139	14.0 [9.0, 33.0]
ICU stay, days, median (IQR)	155	7.0 [5.0, 12.2]
Hospital stay, days, median	150	19.0 [15.0, 31.0]
Acute rejection warranting treatment^a^	155	15.0 (9.7%)
RV function short term^b^	134	4
LV ejection fraction short term^c^	141	63.0 [60.0, 65.0]
RV function long term^b^	76	4
LV ejection fraction long term^d^	75	62.0 [59.5, 65.0]
Number of kidneys transplanted	89	115
Number of livers transplanted	89	50
Pairs of lungs transplanted	89	15

a Moderate rejection defined as Grade 2 R rejection according to the ISHLT (International Society for Heart & Lung Transplantation) Grading score.

b RV function defined on a scale of 1–7 with 1 – normal, 2 – normal-mild, 3 – mild impairment, 4 – mild-moderate impairment, 5 – moderate impairment, 6 – moderate-severe and 7 being severely impaired.

c Short term defined as <6 months. For all data, bar 4 cases this was performed approximately 1 month post-operatively.

d Long term defined as an echo performed after >6 months. For all cases bar 9 this was performed 1 year post-operatively.

### Intraoperative parameters of taNRP

#### Ischaemic times of the donor

The mean withdrawal to reperfusion time was 26.7 min, mean withdrawal to FWIT was 10 min and mean FWIT to reperfusion was 16.7 min. The average cold ischaemic time was 144.2 min (Table 2).

Intra-operative Recipient Characteristics: The recipient pulmonary vascular resistance was 2.6 dynes.

#### Survival & post-transplant outcomes

**Fig. 2:**
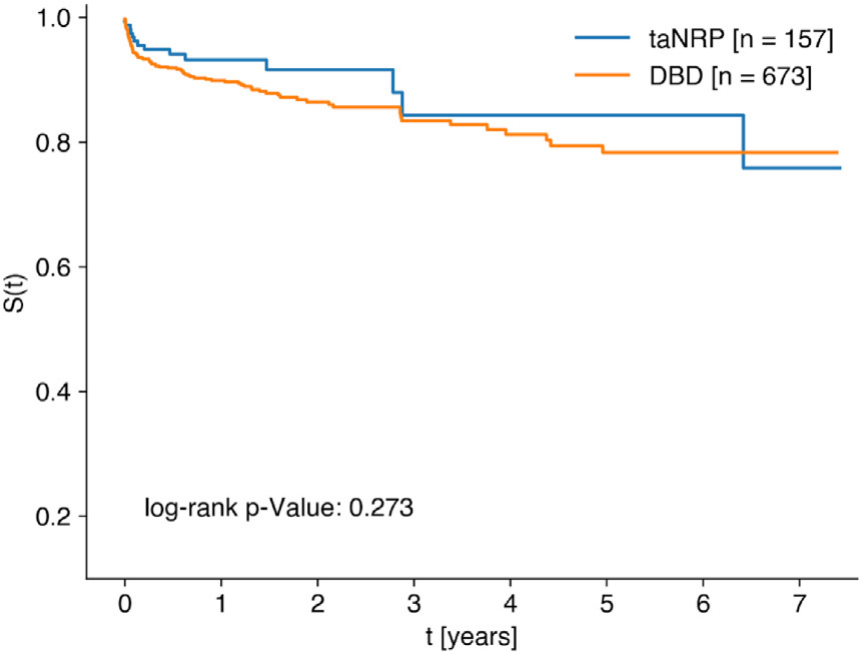
**Kaplan Meier survival in all centres**.

Total cumulative survival after this technique so far has been 247 years. 30-day survival for recipients of ta-NRP hearts was 96.8% (n = 157), 1-year survival was 93.2% (n = 82) and 5-year survival was 84.3% (n = 26) (Fig. 2). 12.8% of patients required postoperative MCS after transplantation. 8.3% (n = 13) of patients required an intra-aortic balloon pump, 5.7% required extra-corporeal membrane oxygenation (ECMO) and no patients required a left ventricular assisted device (LVAD). The median postoperative ventilation duration was 14 h. Median ICU stay was 7 days and median hospital stay was 19 days. Of the 155 patients 9.7% experienced acute rejection warranting treatment. The RV function at 1 month was moderately impaired, with a mean LV ejection fraction of 62%. At 1 year, RV function was still moderately impaired with an LV ejection fraction of 62%.

Of the 89 cases where data was available, 115 kidneys (65%), 50 livers (56%) and 15 (17%) lung transplants were performed.

#### taNRP vs. DBD

The median donor age in the taNRP group (32 years, n = 157) was significantly lower (difference of medians = 4 years) than in the DBD group (36 years, n = 673, p < 0.001). Recipient sex did differ significantly (p = 0.159) between these two groups with 34 recipients (21.7%) in the taNRP group being female compared to 184 recipients (27.3%) in the DBD group. Recipient age did not differ significantly between the two groups (p = 0.073), with a median recipient age of 56 in the taNRP group (n = 157) compared to 54 in the DBD group (n = 673). Cold ischaemic time also differed significantly between the two groups (p < 0.001) with a mean cold ischaemic time of 144.2 min in the taNRP group (n = 136) compared to 174 min in the DBD group (n = 670).

Rates of MCS were very similar between the two groups (p = 1). 12.8% of the patients in the taNRP (n = 20/156) group required MCS compared to 12.7% patients in the DBD group (n = 85/670). 8.3% of patients required an IABP (intra-aortic balloon pump) in the taNRP group (n = 13/156), compared to 2.2% of patients in the DBD group (n = 15/670). This difference was significantly different (p < 0.001). There was a significant difference in VAD (ventricular assisted device) usage post-operatively, with 5.1% of DBD recipients receiving a VAD whilst none of the taNRP patients required a VAD (p < 0.001). ECMO usage was similar in both groups with 5.7% (n = 9/156) taNRP patients requiring ECMO and 5.4% (n = 36/670) of DBD patients requiring ECMO (p = 0.854).

Overall survival did not differ significantly between patients receiving heart transplants with taNRP or DBD, when considering the entire follow-up period (HR = 0.73 [0.41, 1.28], p = 0.273; Table 3). This was also the case when adjusting our model for donor age, recipient age, and cold ischaemic times (HR = 0.96 [0.51, 1.81], p = 0.894).

**Table 3:** Adjusted and unadjusted survival models for taNRP and DBD.

	Hazard ratio [95% CI]	n (taNRP)	n (DBD)	p-value
**Survival (unadjusted)**				
Technique (taNRP vs. DBD)	0.73 [0.41, 1.28]	157	673	0.273
**Survival (adjusted)**				
Technique (taNRP vs. DBD)	0.79 [0.44, 1.40]	157	673	0.414
Donor age (years)	0.99 [0.97, 1.00]	157	673	0.147
Recipient age (years)	1.01 [0.99, 1.02]	157	673	0.464
Donor sex (F)	1.06 [0.67, 1.68]	157	673	0.796
Recipient sex (F)	1.40 [0.88, 2.22]	157	673	0.152

The association between using either CS or ESMP in patients receiving taNRP and survival did not differ significantly between groups (HR = 0.33 [0.06; 1.76], p = 0.196). This was also the case when adjusting the model for donor and recipient ages (HR = 0.3 [0.05, 1.64], p = 0.165).

#### CS vs. ESMP

There was a significant difference in donor age between the CS (n = 136) and ESMP (n = 21) groups (29.5 years vs. 37 years, p = 0.008) (Table 4). There was no significant difference in donor height (p = 0.14), weight (p = 0.85) or ejection faction (p = 0.72). There was also no significant difference in median recipient age (56 vs. 55, p = 0.23). Patient pre-operative pharmacological support was not significantly different between the two group. In the CS group, 63.2% (n = 86) of patients required no pre-operative pharmacological support vs. 55% (n = 11) in the ESMP group. 19.9% of patients required 1 drug in the CS group (n = 27) vs. 40% in the ESMP group (n = 8). 2.9% of patients required 2 drugs pre-operatively in the CS group (n = 4) vs. 5% of patients in the ESMP group (n = 1). 5.9% of patients in the CS group (n = 8) required 3 drugs and 8.1% of patients required ≥4 drugs in the CS group (n = 11), unlike the ESMP group which required no such degree of support.

**Table 4:** Ex situ machine perfusion (ESMP) vs. cold storage during transportation.

	CS	n	ESMP	n	Mean/median difference [95% CI]	p-value
Donor characteristics						
Donor age, median [IQR]	29.5 [23.0, 42.0]	136	35.0 [30.0, 44.0]	21	−5.5 [−12, −2]	0.011
Donor sex, F/M (F%)	23/113 (16.9%)	136	3/18 (14.3%)	21	2.60%	1.0
Height, mean ± SD	174.2 ± 16.7	135	169.2 ± 23.6	21	5.02 [−5.4, 15.5]	0.14
Weight, mean ± SD	81.4 ± 22.4	135	82.9 ± 20.0	21	−1.58 [−10.9, 7.8]	0.85
Ejection fraction, mean ± SD	63.2 ± 6.2	134	64.2 ± 6.9	13	−1.0 [−4.9, 2.9]	0.72
Recipient age, median [IQR]	56.0 [48.0, 63.0]	136	55.0 [43.0, 59.0]	21	1.0 [−2.0, 8.0]	0.23
Recipient sex, F/M (%F)	31/105 (22.8%)	136	3/18 (14.3%)	21	8.50%	0.57
Pharmacological support in the form of vasoactive drugs		136		20		
No pharmacological support	86 (63.2%)		11 (55.0%)		8.2%	0.66
1 drug	27 (19.9%)		8 (40.0%)			
2 drugs	4 (2.9%)		1 (5.0%)			
3 drugs	8 (5.9%)		0 (0.0%)			
≥4 drugs	11 (8.1%)		0 (0.0%)			
Intraoperative parameters						
Mean withdrawal to reperfusion time (SD)	26.9 ± 14.8	133	24.8 ± 11.6	15	2.1 [−4.3, 8.5]	0.71
Mean withdrawal to FTIT (SD)	10.0 ± 13.0	133	9.8 ± 5.9	17	0.2 [−3.3, 3.8]	0.38
Mean FTIT to reperfusion (SD)	16.9 ± 9.4	133	14.9 ± 7.6	15	2.0 [−2.1, 6.2]	0.57
Recipient pulmonary vascular resistance (SD)	2.7 ± 7.1	128	2.0 ± 0.6	16	0.7 [−0.6, 1.9]	0.55
Mean Cold ischaemic time	144.2 ± 78.1	136	n/a^a^	0		–
Mechanical Circulatory Support (MCS) after transplantation (total number of patients, %)	14 (10.3%)	136	6 (28.6%)	21	−18.3%	0.0311
IABP (total number of patients, %)	8 (5.9%)		5 (23.8%)			0.0167
ECMO (total number of patients, %)	8 (5.9%)		1 (4.8%)			1
VAD (total number of patients, %)	–		–			–
Post-transplant outcomes						
Ventilation hours, median [IQR]	13.6 [8.0, 28.5]	120	14.0 [10.5, 38.0]	19	−0.4 [−6.0, 5.0]	0.76
ICU stay, days, median [IQR]	8.0 [5.0, 15.0]	135	5.0 [4.0, 6.0]	21	3.0 [1.0, 5,0]	0.002
Hospital stay, days, median [IQR]	19.0 [15.0, 31.0]	129	20.0 [17.0, 26.0]	21	−1 [−4, 4]	0.77
Acute rejection warranting treatment	13.0 (9.7%)	134	2.0 (9.5%)	21	0.1%	1
Number of other organs transplanted						
Kidneys	76	68	39	21		
Livers	32	68	18	21		
Pairs of Lungs	13	68	2	21		
Heart function post-transplant						
LV Ejection Fraction post-op ST, median [IQR]^c^	65.0 [60.0, 65.0]	120	60.0 [55.0, 63.0]	21	5.0 [0.0, 7.0]	0.06
LV Ejection Fraction post-op LT, median [IQR]^d^	63.5 [60.0, 65.0]	56	60.0 [54.5, 63.0]	19	3.5 [0.0, 6.0]	0.034
RV function post-op ST, median [IQR]^c^	4.0 [3.0, 4.0]	113	3.0 [2.5, 3.0]	21	b	
RV function post-op LT, median [IQR]^d^	4.0 [4.0, 4.0]	57	3.0 [3.0, 3.0]	19	b	

a Note that ESMP reperfuses the heart with warm, oxygenated blood and therefore the term ‘cold ischaemic time’ is not applicable.

b RV function defined on a scale of 1–7 with 1 – normal, 2 – normal-mild, 3 – mild impairment, 4 – mild-moderate impairment, 5 – moderate impairment, 6 – moderate-severe and 7 being severely impaired. Note that a difference has not been calculated as this is not a qualitative score.

c Short term defined as <6 months. For all data, bar 4 cases this was performed approximately 1 month post-operatively.

d Long term defined as an echo performed after >6 months. For all cases bar 9 this was performed 1 year post-operatively.

The intraoperative parameters did not differ significantly between the 2 groups. Mean WSLT to reperfusion time was 26.9 min in the CS group and 24.8 min in the ESMP group (p = 0.71) The mean withdrawal to FWIT was 10 min in the CS group and 9.8 min in the ESMP group (p = 0.38). FWIT to reperfusion was 16.9 min in the CS group and the ESMP group it was 14.9 min (p = 0.57). The mean recipient pulmonary vascular resistance was 2.7 mmHg min/l in the CS group and 2.0 mmHg min/l in the ESMP group (p = 0.55).

The use of MCS early after surgery differed significantly between the two groups (p = 0.0311), due specifically to the increased rate of IABP utilisation (p = 0.0167) in the ESMP group. 10.3% of patients in the CS group required MCS (n = 14.0), compared to 28.6% in the ESMP group (n = 6). Of these patients in the CS group 5.9% (n = 8) required an IABP and 5.9% (n = 8) required ECMO. In the ESMP group 23.8% of patients (n = 5) required an intra-aortic balloon pump (IABP) and 4.8% (n = 1) of patients required extra-corporeal membrane oxygenation (ECMO) and no patients required the use of a LVAD.

The median time spent on a ventilator in the CS group was 13.6 h (n = 120) and did not differ significantly compared to the 14 h in the ESMP group (n = 19) (p = 0.76). ICU stay did differ significantly between the two groups (p = 0.002). The median ICU stay in the CS group was 8 days (n = 135) compared to 5 days (n = 21) in the ESMP group. Hospital stay did not differ significantly between the two groups (p = 0.77). The median hospital stay in the CS group was 19 days (n = 129) compared to 20 days in the ESMP group (n = 21). Acute rejection warranting treatment was practically identical in both groups with 9.7% of patients in the CS group (n = 13) experiencing this, compared to 9.5% of patients (n = 2) in the ESMP group (p = 1).

LV ejection fraction (LVEF) post-transplant in the short term tended towards significance between the two groups with possible superiority in the CS group. LVEF in the short term was 65% (n = 120) compared to 60% (n = 21) (p = 0.06). Median LVEF in the long term did differ significantly between the two groups (p = 0.034), with superior LVEF in the CS group (63.5%, n = 56) when compared to the ESMP group (60%, n = 19). RV function short-term was mild-moderately impaired in the CS group (n = 4.0) compared to mildly impaired in the ESMP group (n = 21). In the long term, RV function was again mild-moderately impaired in the CS group (n = 57) and was mildly impaired in the ESMP group (n = 19).

Causes of death in the taNRP and DBD groups are shown in Table 5.

**Table 5:** Causes of death in taNRP and DBD groups.

Cause of death	taNRP			DBD		
	n	Proportion of deaths	Proportion of cases	n	Proportion of deaths	Proportion of cases
CAV	2	14.3%	1.3%	4	4.5%	0.6%
Acute rejection	–	–	–	11	12.4%	1.6%
Lymphoma	1	7.1%	0.6%	–	–	–
Malignancy (other)	–	–	–	2	2.2%	0.3%
Infection, including sepsis (non-CMV)	5	35.7%	3.2%	15	16.9%	2.2%
Primary graft failure	–	–	–	21	23.6%	3.1%
Technical	1	7.1%	0.6%	2	2.2%	0.3%
Multiple organ failure	2	14.3%	1.3%	9	10.1%	1.3%
Cerebrovascular	1	7.1%	0.6%	4	4.5%	0.6%
Pulmonary	1	7.1%	0.6%	10	11.2%	1.5%
Other	1	7.1%	0.6%	11	12.4%	1.6%
Total	**14**	**100%**	**8.9%**	**89**	**100%**	**13**.**2%**

## Discussion

Worldwide there is a shortage of heart donors, resulting in considerable waitlist mortality and missed improvement in quality of life in patients who do not receive a heart. It has been estimated that DCD heart transplantation can increase the heart donor pool by about 30%.^3^ taNRP is a method of in-situ preservation of the DCD heart and therefore has the potential to further increase the number of hearts transplanted and reduce waitlist times and mortality. An added value is that it allows to simultaneously preserve several organs, without the need of several organ-specific ex situ machine perfusion devices. This decreases complexity and costs. In addition, in-situ preservation in DCD transplantation has demonstrated improved organ utilisation in abdominal transplantation.^12^

DBD transplantations performed in the same timeframe in each centre served as a comparison group, as it represents the ‘gold-standard’ for heart transplantation, as it is a widely performed technique and one that all the centres in this study have considerable experience with. In this study it was not possible to compare taNRP to DPP, given that most centres do not perform the latter.

taNRP demonstrated comparable overall survival to DBD heart transplantation. It may be possible that taNRP offers a protective effect on the heart when compared to DBD transplantation, reflected in increased survival at earlier follow-up timepoints, however, any such claim is difficult to support in the absence of a randomised controlled trial. taNRP offers protection of the DCD heart by minimising ischaemic times. The median FWIT was 16.7 min. This is significantly less than the 30 min of ischaemia associated with permanent damage to myocytes.^24^ However, DBD has no ischaemic time during this phase of procurement, therefore the question remains as to why taNRP may protect the graft in the short term. This may be due to the absence of a Cushing's reflex,^25^ caused by the release of catecholamines during brainstem death and the probable transient resulting Takotsubo-like cardiomyopathy, or ischaemic preconditioning. Whatever the cause, it is not possible to confidently claim that taNRP offers superior survival to DBD, however the results are encouraging so far. It is also worth mentioning that in our Cox model, the hazard ratio comparing survival for taNRP and DBD showed that donor age, recipient age, donor sex and recipient sex had negligible effects on survival. Nevertheless, we note that taNRP donors were considerably younger than DBD donors, and that median ischaemic times were also reduced in the taNRP group. We chose to focus on these two parameters as they are the single most influential parameters on transplant survival.^26^ It is worth clarifying that this was not a controlled trial, therefore it is possible that there could be confounders.

Given that survival was similar between the two groups, but the cold ischaemic time was significantly shorter in the taNRP group and given the fact that the hazard ratio was 1.00 for cold ischaemic times, it suggests that in this study cold ischaemic time was not a significant contributing factor to mortality. This is most likely due to the relatively short cold ischaemic times in the transplants included in this study.

It is interesting to note the distribution of the causes of death. None of the 14 deaths in the taNRP group were due to PGD compared to 23% of deaths in the DBD group. However, given that overall survival was similar between the two groups and the rates of MCS (Table 1) was practically identical in both groups it seems as if there is no significant difference in the rates of PGD between taNRP and DBD hearts.

Rates of MCS utilisation were practically identical between the taNRP and DBD groups. However, it is worth noting that there were differences in the kind of MCS used in each group. ECMO utilisation was similar between both groups. taNRP recipients had an almost four-fold higher rate of IABP utilisation post transplantation compared to the DBD group. However, not a single taNRP recipient required a VAD compared to 5.1% of DBD recipients. VADs are a significantly more invasive and expensive method of MCS compared to IABPs. It is difficult to comment on the exact reasons for this. It could be due to the potentially protective effects mentioned above, i.e. preconditioning and the lack of a Cushing's reflex. It is worth noting that there are confounders and therefore these results should be interpreted with caution. There is a need for a randomised control trial to investigate whether taNRP does in fact have lower rates of VAD usage than other forms of transplantation. Median LV function in the short-term (typically within 1 month of the operation) was normal at 63%. RV function was mild-moderately impaired. At 1-year post-transplant the echocardiography showed median LV function to be at 62% and RV function still mild-moderately impaired. This suggests that these hearts maintain healthy function at 1-year post-transplantation.

An important consideration for the use of taNRP is whether the hearts are preserved by ESMP or CS in the interim between procurement and transplantation. Our data demonstrates that there is no significant difference in survival between the two groups. There was a significant difference however in the median ICU stays, being significantly shorter in the ESMP group (5 vs. 8 days) when compared to CS. This was despite the median age of the ESMP donor being 8 years older than the CS donor. In addition to this, there was superior RV function in the ESMP group, with RV function only being mildly impaired instead of mild-moderately impaired as in the CS group. However, it is worth noting that each centre used their own criteria to define mild, moderate and severe dysfunction. Whilst one would expect these criteria to be fairly similar, it does not make any valid comparisons possible between ESMP and CS. On the other hand, the CS group had a statistically significantly better LV function at 1-year post-transplant, however the difference was probably within the range of error of the tools for estimating ejection fraction. Besides this, there was also a significantly lower utilisation of post-transplant MCS in the CS group, specifically, the use of IABP. This difference may be due to the small size of the ESMP group or due to centre differences in utilising the IABP. Alternatively, recent pig work has demonstrated that heart function during ESMP declines steadily over time. Over a period of 8 h, pig hearts preserved with ESMP demonstrated deranged metabolomics as well as both systolic and diastolic dysfunction.^27^ Importantly, 2 human hearts also demonstrated an impaired metabolic state. Given that nearly all ESMP was done in one centre (19 of 21 in Papworth, UK), it is very possible that differences are due to centre bias. Overall, these data suggest that there is no clinically significant difference in outcomes between CS and ESMP.

It may be that in taNRP there is no need for ESMP as the replenishment of glucose and reoxygenation follow in-situ reperfusion. taNRP allows for the conversion of a non-heart beating donor to a heart beating donor similar and therefore does not require ESMP, much like DBD transplantation.^28^ These results indicate a great financial benefit of taNRP when compared to DPP, given that the consumable used on a single donor run cost in the order of $75,000 on top of the $400,000 for the perfusion machine, significantly more expensive than the use of CS. Furthermore, as previously mentioned NRP improves outcomes and utilisation of abdominal organs.^10^, ^11^, ^12^, ^13^ This is associated with further cost-savings and improved post-transplant outcomes. However, we have not performed a full economic analysis of the long-term costs of taNRP compared to DPP. Nevertheless, the per run cost savings are very encouraging and should be explored further in the context of a randomised clinical trial.

Moreover, taNRP enables in-situ assessment of heart function. This is a major advantage over DPP, as currently, one of the main indicators used for assessing ex-situ heart function are lactate levels.^29^ This is based off a single abstract with just 49 patients in DBD transplantation. More recent work has demonstrated that lactate levels do not correlate with primary graft dysfunction and that lactate is not a suitable marker for heart function.^30^ It is worth noting that other methods of assessing heart function during DPP include rate, rhythm, coronary flow rate and aortic pressure but they are not effective markers of heart function and do not compare to in-situ analysis of heart function nor have they been correlated to long term survival. This leaves DPP more expensive and without any suitable markers of donor organ ‘transplantability’. These issues do not exist in taNRP transplantation. Therefore, taNRP may be more financially viable and enables assessment of in situ cardiac function. These together suggest that taNRP may be preferable to DPP.

It is difficult to comment on the utilisation of other organs. The study was not designed to compare utilisation. Furthermore, outcomes of the transplanted organs are also unavailable. However, the current utilisation of lungs seems to be low. The reason for this is not known. However, we believe that this arises from a misconception about the quality of taNRP DCD lungs. Animal work has demonstrated that lung function in taNRP donors is preserved.^31^ The issue of lung utilisation must be addressed in the context of a randomised control trial.

This study has several strengths. It is the largest, international, multi-centre observational study on taNRP to date. There were 157 cases of taNRP until July 29th and 673 cases of DBD transplantation in this study. The utilisation of DBD cohorts from the centres in this study from the same period as DBD has enabled comparisons to be made between the two techniques. Moreover, this study contributes to a limited literature on taNRP, where so far there have only been 7 centres reporting on 53 cases between them.^14^, ^15^, ^16^, ^17^, ^18^, ^19^, ^20^

The study had several limitations. The first is the shorter median follow-up time, especially from three years onwards where the number of censored events increases considerably. This was unavoidable given that world-wide there have been a limited number of cases that were done more than 3 years ago. Another issue with the study is that site is a confounding factor. There were large variations between taNRP and DBD survival between centres. Further to this, given the lack of demographic and other data not collected, it is difficult to assess centre-based bias. Moreover, the majority of ESMP (19/21) were performed in one centre, therefore it is not possible to definitively say whether differences between CS and ESMP groups were due to centre differences or these differences in technique. Further to this, the sample distribution was not equal between CS and ESMP groups, with CS having significantly more cases. It also would have been beneficial to analyse the pre-transplantation use of MCS in recipients, as this is also an important factor in influencing post-transplant survival,^26^ but unfortunately these data were not available. Further to this, gathering data from this number of centres is a sensitive task and some centres were unable to provide a full characterisation of their DBD data. Lastly, we also acknowledge that in some cases data are missing of certain parameters.

Increased acceptance and utilisation of ta-NRP for DCD heart retrieval will require studies demonstrating the absence of cerebral perfusion during taNRP. A recent paper has highlighted several rapid and effective methods of ensuring no cerebral blood flow, although these have yet to be proven clinically.^32^ In addition, we believe future work should take the form of a randomised control trial to better understand the outcomes of taNRP in comparison to DBD transplantation.

Finally, we can reasonably conclude that taNRP increases the donor pool significantly. In this study the technique increased the number of heart transplantations performed by 23%. One centre also performed an additional 85 cases by DPP. When these are included, the DCD transplantation activity in these centres have cumulatively increased activity by 36%. This is particularly exciting given the novelty of the technique and the relative lack of experience that most centres have with it in this early stage of its clinical use. This has an immense prognostic effect for individuals who are waiting on the heart transplant list. Both in terms of their quality of life and their survival as they are more likely to get a heart. This is exactly the kind of innovation needed to treat the issue of ever-growing rates of ESHF.

## Contributors

*Louca J* – primary author – collected data, performed data analysis, verified underlying data and wrote the paper. *Öchsner M* – data analysis and wrote the paper. *Shah A* – initial acquisition of data, data collection and commented on the paper. *Hoffman J* – initial acquisition of data, data collection and commented on the paper. *Debose-Scarlett A* – initial acquisition of data, data collection, verified underlying data and commented on the paper. *González Vilchez-F* – data collection and commented on paper. *Garrido I* – data collection and commented on paper. *Royo-Villanova M* – data collection and commented on paper. *Domínguez-Gil B* – data collection, aided in study design, verified underling data and commented on paper. *Deane S* – initial acquisition of data, data collection and commented on the paper. *James L* – initial acquisition of data, data collection, verified underlying data and commented on the paper. *Moazami N* – initial acquisition of data, data collection and commented on the paper. *Rega F* – initial acquisition of data, data collection and commented on the paper. *Brouckaert J* – data collection, verified underlying data and commented on paper. *Van Cleemput J* – data acquisition and commented on paper. *Tchana-Sato V* – initial acquisition of data, data collection, verified underlying data and commented on the paper. *Diawara Bandiougou* – initial acquisition of data, data collection and commented on paper. *Urban M* – initial acquisition of data, data collection, verified underlying data and commented on the paper. *Manara A* – reviewed and commented on the paper, initial design of study. *Berman M* – initial acquisition of data, reviewed and commented on the paper. *Messer S* – reviewed and commented on paper, contributed to initial conception of study. *Large S* – Reviewed and commented on the paper, Initial conception of the study. WISPG – Members of WISPG were involved in data acquisition, data collection and reviewing the paper. All authors had full access to the data and agreed to submit the paper for publication.

## Data sharing statement

Deidentified patient data may be made available by contacting the corresponding author after publication. Data will be shared with investigator support, after approval of a proposal. For data enquiries please contact jol20@cam.ac.uk.

## Declaration of interests

None of the authors has a financial relationship with a commercial entity that has an interest in the subject of the presented manuscript or other conflicts of interest to disclose.

## Appendix A Supplementary data

Appendix Tables S1–S4

STROBE Checklist

Ethics Approval

Full Author List
