# Supplementary material for: The international experience of in-situ recovery of the DCD heart: a multicentre retrospective observational study
Source: eClinicalMedicine. 2023 Mar 2;58:101887. doi: 10.1016/j.eclinm.2023.101887 (PMC9995283; doi:10.1016/j.eclinm.2023.101887)
Supplement: Appendix Tables S1–S4 [file mmc1.pdf]

Final Appendix

Supplementary Information

List of Centres Involved
Royal Papworth Hospital UK, Vanderbilt University Medical Center, University Nebraska Medical Centre & New York University, USA, The University Hospitals of Leuven & Liege, Belgium and Complexo Hospitalario Universitario A Coruña, Hospital Clinic Universitari, Hospital Universitario Virgen de la Arrixaca, Hospital General Universitario Gregorio Marañón, Hospital Universitari de Bellvitge, Hospital Universitario La Paz, Hospital Universitario Marqués de Valdecilla, Hospital Universitario de Gran Canaria Doctor Negrín & Hospital Universitario Puerta de Hierro-Majadahonda, Spain.

Method of cannulation
Centres in the UK and US performed post-mortem central cannulation, with cannulae placed in the aorta and right atrium. Centres in Belgium and Spain performed peripheral cannulation where the femoral artery and femoral vein were cannulated instead. More details on the methods of operation can be found here[14-20].

Results
Donor characteristics of taNRP & DBD groups.
The median donor age was 32 years (IQR = 23-43). Of the 157 taNRP donors 26 (16.6%) were female and 131 were male (83.4%). 226 (33.6%) of donors were female in the DBD group and 447 (63.4%) were male. This difference was significant between the two groups (p=0.001). The mean height was 173·6 cm and mean weight was 81·6 kg in the taNRP group. The mean LV ejection fraction at the time of donation, (before WSLT) was 63·3% (n = 147). 62·2% of patients were on no pre-operative pharmacological support in the form of vasoactive drugs, 22·4% were on 1 drug, 3·2% were on 2 drugs, 5·1% were on 3 drugs and 7·1% > = 4 drugs.

Intraoperative Parameters of taNRP
Ischaemic Times of the Donor
The mean withdrawal to reperfusion time was 26·7 minutes, mean withdrawal to FWIT was 10 minutes and mean FWIT to reperfusion was 16·7 minutes. The average cold ischaemic time was 144·2 minutes.

Intra-operative Recipient Characteristics:
The recipient pulmonary vascular resistance was 2·6 dynes.

Survival & post-transplant outcomes
Total cumulative survival after this technique so far has been 247 years. 30-day survival for recipients of ta-NRP hearts was 96·8% (n = 157), 1-year survival was 93·2% (n = 82) and 5-year survival was 84·3% (n = 26) (Figure 2). 12·8% of patients required postoperative MCS after transplantation. 8·3% (n=13) of patients required an intra-aortic balloon pump, 5·7% required extra-corporeal membrane oxygenation (ECMO) and no patients required a left ventricular assisted device (LVAD). The median postoperative ventilation duration was 14 hours. Median ICU stay was 7 days and median hospital stay was 19 days. Of the 155 patients 9·7% experienced acute rejection warranting treatment. The RV function at 1 month was moderately impaired, with a mean LV ejection fraction of 62%. At 1 year, RV function was still moderately impaired with an LV ejection fraction of 62%.

Of the 89 cases where data was available, 115 kidneys (65%), 50 livers (56%) and 15 (17%) lung transplants were performed.

taNRP vs DBD
The median donor age in the taNRP group (32 years, n = 157) was significantly lower (difference of medians = 4 years;) than in the DBD group (36 years, n = 673, p<0·001). Recipient sex did differ significantly (p=0.159) between these two groups with 34 recipients (21.7%) in the taNRP group being female compared to 184 recipients (27.3%) in the DBD group. Recipient age did not differ significantly between the two groups (p = 0·073), with a median recipient age of 56 in the taNRP group (n = 157) compared to 54 in the DBD group (n = 673). Cold ischaemic time also differed significantly between the two groups (p<0·001) with a mean cold ischaemic time of 144·2 minutes in the taNRP group (n = 136) compared to 174 minutes in the DBD group (n = 670).

Rates of MCS were very similar between the two groups (p=1). 12.8% of the patients in the taNRP (n=20/156) group required MCS compared to 12.7% patients in the DBD group (n=85/670). 8.3% of patients required an IABP in the taNRP group (n=13/156), compared to 2.2% of patients in the DBD group (n=15/670). This difference was significantly different (p<0.001). There was a significant difference in VAD usage post-operatively, with 5.1% of DBD recipients receiving a VAD whilst none of the taNRP patients required a VAD (p<0.001). ECMO usage was similar in both groups with 5.7% (n=9/156) taNRP patients requiring ECMO and 5.4% (n=36/670) of DBD patients requiring ECMO (p=0.854).

Overall survival did not differ significantly between patients receiving heart transplants with taNRP or DBD, when considering the entire follow-up period (HR = 0·73 [0·41, 1·28], p = 0·273). This was also the case when adjusting our model for donor age, recipient age, and cold ischaemic times (HR = 0·96 [0·51, 1·81], p = 0·894).

The association between using either CS or ESMP in patients receiving taNRP and survival did not differ significantly between groups (HR = 0·33 [0·06; 1·76], p = 0·196). This was also the case when adjusting the model for donor and recipient ages (HR = 0·3 [0·05, 1·64], p = 0·165).

CS vs ESMP

There was a significant difference in donor age between the CS (n=136) and ESMP (n=21) groups (29·5 years vs 37 years, p = 0·008). There was no significant difference in donor height (p = 0·14), weight (p = 0·85) or ejection faction (p = 0·72). There was also no significant difference in median recipient age (56 vs 55, p = 0·23). Patient pre-operative pharmacological support was not significantly different between the two group. In the CS group, 63·2% (n = 86) of patients required no pre-operative pharmacological support vs 55% (n = 11) in the ESMP group· 19·9% of patients required 1 drug in the CS group (n = 27) vs 40% in the ESMP group (n = 8). 2·9% of patients required 2 drugs pre-operatively in the CS group (n = 4) vs 5% of patients in the ESMP group (n = 1). 5·9% of patients in the CS group (n = 8) required 3 drugs and 8·1% of patients required > = 4 drugs in the CS group (n = 11), unlike the ESMP group which required no such degree of support.

The intraoperative parameters did not differ significantly between the 2 groups. Mean WSLT to reperfusion time was 26·9 minutes in the CS group and 24·8 minutes in the ESMP group. (p = 0·71) The mean withdrawal to FWIT was 10 minutes in the CS group and 9·8 minutes in the ESMP group (p = 0·38). FWIT to reperfusion was 16·9 minutes in the CS group and the ESMP group it was 14·9 minutes (p = 0·57). The mean recipient pulmonary vascular resistance was 2·7 mmHg·min/l in the CS group and 2·0 mmHg·min/l in the ESMP group (p = 0·55).

The use of MCS early after surgery differed significantly between the two groups (p = 0·0311), due specifically to the increased rate of IABP utilisation (p=0·0167) in the ESMP group. 10·3% of patients in the CS group required MCS (n = 14·0), compared to 28·6% in the ESMP group (n = 6). Of these patients in the CS group 5·9% (n=8) required an IABP and 5·9% (n=8) required ECMO. In the ESMP group 23·8% of patients (n = 5) required an intra-aortic balloon pump (IABP) and 4·8% (n = 1) of patients required extra-corporeal membrane oxygenation (ECMO) and no patients required the use of a LVAD.

The median time spent on a ventilator in the CS group was 13·6 hours (n = 120) and did not differ significantly compared to the 14 hours in the ESMP group (n = 19) (p = 0·76). ICU stay did differ significantly between the two groups (p = 0·002). The median ICU stay in the CS group was 8 days (n = 135) compared to 5 days (n = 21) in the ESMP group. Hospital stay did not differ significantly between the two groups (p = 0·77). The median hospital stay in the CS group was 19 days (n = 129) compared to 20 days in the ESMP group (n = 21). Acute rejection warranting treatment was practically identical in both groups with 9·7% of patients in the CS group (n = 13) experiencing this, compared to 9·5% of patients (n = 2) in the ESMP group (p = 1).

LV ejection fraction (LVEF) post-transplant in the short term tended towards significance between the two groups with possible superiority in the CS group. LVEF in the short term was 65% (n = 120) compared to 60% (n = 21) (p = 0·06). Median LVEF in the long term did differ significantly between the two groups (p = 0·034), with superior LVEF in the CS group (63·5%, n = 56) when compared to the ESMP group (60%, n = 19). RV function short-term was mild-moderately impaired in the CS group (n = 4·0) compared to mildly impaired in the ESMP group (n = 21). In the long term, RV function was again mild-moderately impaired in the CS group (n = 57) and was mildly impaired in the ESMP group (n = 19).

Full Author List

John Louca[1] BA,
Marco Öchsner[1] BSc,
Ashish Shah[2] MD,
Jordan Hoffman[2] MD,
Francisco González Vilchez[3] PhD,
Iris Garrido[4] PhD,
Mario Royo-Villanova[4] PhD,
Beatriz Domínguez-Gil[5] PhD,
Deane Smith[6] MD,
Leslie James[6] MD,
Nader Moazami[6] MD,
Filip Rega[7] PhD,
Janne Brouckaert[7] MD,
Johan Van Cleemput[7] PhD,
Katrien Vandendriessche[7] MD,
Vincent Tchana-Sato[8] PhD,
Bandiougou Diawara[8] MD,
Marian Urban[9] PhD,
Alex Manara[10] MB BCh,
Marius Berman[11] MD,
Simon Messer[12] PhD,
Stephen Large [11] MD,

Authors in the study group – the WISP Group

First Name	Surname
Nirav	Patel
Rohan	Sanghera
Constantinos	Kapetanos
Antonio	Rubino
Sai	Bhagra
Luis-Alberto	Martinez-Marin
Jordan	Allen
Chindu	John
Daniel	Normington
Steven	Tsui
Aravinda	Page
Vanessa	Chow
William	McMaster
Alicia	Pérez-Blanco
Elisabeth	Torres
José	Cuenca
Fernando	Mosteiro

Marta	Farrero
Elena	Sandoval
Manuela	Camino
Juan	Jáurena
Fabrizio	Sbraga
Eva	Oliver
Antonio	Quintana
Vincente	Morant
Belen	Estébanez
Álvaro	Rocafort
Manuel	Cobo
Francisco	Nistal
Manuel	Gómez-Bueno
Marina	Pérez-Redondo
Arne	Neyrinck
Diethard	Monbaliu
Laurens	Ceulemans

Supplementary Figures

Table 1. Point Estimate Survival of taNRP compared to DBD recipients (supplementary information)

timepoint	DBD	taNRP	t-statistic	p-value
1 month	0·945	0·968	1·29	0·26
1 year	0·899	0·932	1·50	0·22
2 years	0·864	0·916	2·18	0·14
3 years	0·834	0·843	0·02	0·88
4 years	0·812	0·843	0·24	0·63
5 years	0·783	0·843	0·77	0·38

Table 2. Adjusted model of survival including cold ischaemic times (i.e. excluding the ESMP group)

survival (adjusted)	Hazard ratio [95%CI]	n (taNRP)	n (DBD)	p-Value
technique (taNRP vs. DBD)	0·97 [0·51, 1·83]	136	670	0·921
donor age (years)	0·99 [0·97, 1·00]	136	670	0·128
recipient age (years)	1·01 [0·99, 1·02]	136	670	0·454
donor sex (F)	1·10 [0·69, 1·73]	136	670	0·696
recipient sex (F)	1·41 [0·88, 2·23]	136	670	0·149
cold ischaemic time (min)	1·00 [1·00, 1·00]	136	670	0·382

Table 3. Survival in the CS group vs ESMP group (supplementary information)

Time (years)	0	1	2	3	4	5	6	7
CS [n = 136]								
At risk	135	53	17	6	5	3	3	1
Censored	0	73	108	119	120	122	122	123
Events	1	10	11	11	11	11	11	12
ESMP [n = 21]								
At risk	21	19	18	16	14	10	9	4
Censored	0	2	3	3	5	9	10	15
Events	0	0	0	2	2	2	2	2

Table 4. Causes of death in the taNRP group (supplementary information)

**Case**	**Recipient Age (years)**	**Use of MCS post-transplant**	**Duration of survival (days)**	**Causes of Death**
**6**	64	No	2344	CAV
**20**	64	No	1017	COVID-19
**21**	50	No	1053	CAV
**41**	54	No	0	Intraoperative aortic dissection at time of cannulation
**65**	65	No	23	Sepsis
**96**	68	No	230	Lymphoma
**97**	54	No	539	Pneumonia after massive stroke
**98**	56	No	36	COVID-19
**105**	67	ECMO	49	Infection
**108**	70	ECMO	75	Infection
**136**	49	No	4	Multi-organ failure
**137**	2 months	No	172	Infection
**146**	44	No	28	Haemorrhage
**147**	50	ECMO	20	Multi-organ failure
