# Supplementary material for: The international experience of in-situ recovery of the DCD heart: a multicentre retrospective observational study
Source: eClinicalMedicine. 2023 Mar 2;58:101887. doi: 10.1016/j.eclinm.2023.101887 (PMC9995283; doi:10.1016/j.eclinm.2023.101887)
Supplement: Full Author List [file mmc4.docx]

Full Author List

John Louca^1^ BA,

Marco Öchsner^1^ BSc,

Ashish Shah^2^ MD,

Jordan Hoffman^2^ MD,

Francisco González Vilchez^3^ PhD,

Iris Garrido^4^ PhD,

Mario Royo-Villanova^4^ PhD,

Beatriz Domínguez-Gil^5^ PhD,

Deane Smith^6^ MD,

Leslie James^6^ MD,

Nader Moazami^6^ MD,

Filip Rega^7^ PhD,

Janne Brouckaert^7^ MD,

Johan Van Cleemput^7^ PhD,

Katrien Vandendriessche^7^ MD,

Vincent Tchana-Sato^8^ PhD,

Bandiougou Diawara^8^ MD,

Marian Urban^9^ PhD,

Alex Manara^10^ MB BCh,

Marius Berman^11^ MD,

Simon Messer^12^ PhD,

Stephen Large ^11^ MD,

Authors in the study group – the WISP Group

| First Name | Surname |
| --- | --- |
| Nirav | Patel |
| Rohan | Sanghera |
| Constantinos | Kapetanos |
| Antonio | Rubino |
| Sai | Bhagra |
| Luis-Alberto | Martinez-Marin |
| Jordan | Allen |
| Chindu | John |
| Daniel | Normington |
| Steven | Tsui |
| Aravinda | Page |
| Vanessa | Chow |
| William | McMaster |
| Alicia | Pérez-Blanco |
| Elisabeth | Torres |
| José | Cuenca |
| Fernando | Mosteiro |
| Marta | Farrero |
| Elena | Sandoval |
| Manuela | Camino |
| Juan | Jáurena |
| Fabrizio | Sbraga |
| Eva | Oliver |
| Antonio | Quintana |
| Vincente | Morant |
| Belen | Estébanez |
| Álvaro | Rocafort |
| Manuel | Cobo |
| Francisco | Nistal |
| Manuel | Gómez-Bueno |
| Marina | Pérez-Redondo |
| Arne | Neyrinck |
| Diethard | Monbaliu |
| Laurens | Ceulemans |
