# Supplementary material for: The international experience of in-situ recovery of the DCD heart: a multicentre retrospective observational study
Source: eClinicalMedicine. 2023 Mar 2;58:101887. doi: 10.1016/j.eclinm.2023.101887 (PMC9995283; doi:10.1016/j.eclinm.2023.101887)
Supplement: Ethics Approval [file mmc3.pdf]

NHS
Health Research Authority

NRES Committee East of England - Cambridge East

The Old Chapel
Royal Standard Place
Nottingham
NG1 6FS

Tel: 0115 8839390

28 May 2014

Dr Sarah Fielding
Research Office: Cardiology and Cardiac Surgery
Papworth Hospital NHS Foundation Trust
Papworth Everard
Cambridgeshire

Dear Dr Fielding

**Study title:**	**Is the non heart beating donor a suitable source for clinical heart transplantation?**
**REC reference:**	**05/Q0104/111**
**EudraCT number:**	**N/A**
**Amendment number:**	**Substantial Amendment 08**
**Amendment date:**	**16 May 2014**
**IRAS project ID:**	

The above amendment was reviewed by the Sub-Committee in correspondence.

Ethical opinion

The members of the Committee taking part in the review gave a favourable ethical opinion of the amendment on the basis described in the notice of amendment form and supporting documentation.

Approved documents

The documents reviewed and approved at the meeting were:

Document	*Version*	*Date*
Covering letter on headed paper	Signed by Dr Sarah Fielding	
Notice of Substantial Amendment (non-CTIMP)	Substantial Amendment 08	16 May 2014
Participant information sheet (PIS) [Family Information Sheet]	5	24 May 2013
Participant information sheet (PIS) [Family Information Sheet]	Clean and tracked changes version 6	09 May 2014

Membership of the Committee

The members of the Committee who took part in the review are listed on the attached sheet.

R&D approval

All investigators and research collaborators in the NHS should notify the R&D office for the relevant NHS care organisation of this amendment and check whether it affects R&D approval of the research.

Statement of compliance

The Committee is constituted in accordance with the Governance Arrangements for Research Ethics Committees and complies fully with the Standard Operating Procedures for Research Ethics Committees in the UK.

We are pleased to welcome researchers and R & D staff at our NRES committee members' training days – see details at http://www.hra.nhs.uk/hra-training/

**05/Q0104/111:**	**Please quote this number on all correspondence**

Yours sincerely

pp C Halliwell

Reverend Keith Lackenby
Chair (co-opted)

E-mail: NRESCommittee.EastofEngland-CambridgeEast@nhs.net

Enclosures: *List of names and professions of members who took part in the review*

Copy to: *Mr Stephen Large, Papworth Hospital NHS Trust,*

NRES Committee East of England - Cambridge East

Attendance at Sub-Committee of the REC meeting in correspondence

Committee Members:

Name	*Profession*	*Present*	*Notes*
Dr Madhu Davies	Consultant in Pharmaceutical Medicine and Devices	Yes	
Reverend Keith Lackenby (Chair – co-opted)	Lay member	Yes	

Also in attendance:

Name	*Position (or reason for attending)*
Ms Carolyn Halliwell	REC Assistant
